# The Effect of Maternal Age at the First Childbirth on Gestational Age and Birth Weight: The Japan Environment and Children’s Study (JECS)

**DOI:** 10.2188/jea.JE20170283

**Published:** 2019-05-05

**Authors:** Hyo Kyozuka, Keiya Fujimori, Mitsuaki Hosoya, Seiji Yasumura, Tadahiko Yokoyama, Akiko Sato, Koichi Hashimoto, the Japan Environment and Children’s Study (JECS) Group

**Affiliations:** 1 Fukushima Regional Center for the Japan Environmental and Children’s Study, Fukushima, Japan; 2 Department of Obstetrics and Gynecology, School of Medicine, Fukushima Medical University, Fukushima, Japan; 3 Department of Pediatrics, School of Medicine, Fukushima Medical University, Fukushima, Japan; 4 Department of Public Health, School of Medicine, Fukushima Medical University, Fukushima, Japan

**Keywords:** maternal age, preterm birth, first delivery, low birth weight, birth cohort study

## Abstract

**Background:**

In Japan, mean maternal age at first childbirth is increasing. The aim of this study was to investigate whether maternal age at the first childbirth is a risk factor for preterm birth (PTB), low birth weight (LBW), and small for gestational age (SGA).

**Methods:**

We used the results of Japan Environment and Children’s Study (JECS) who gave birth in 2011–2014. Cases of primiparous singleton pregnancies where the subject was ≥20 years and delivered after 22 weeks were included. All subjects were categorized into five groups according to maternal age: 20–24, 25–29, 30–34, 35–39, and ≥40 years. Adjusted odds ratios (aORs) for PTB (before 37 and 34 weeks), LBW (<2,500 g and <1,500 g), and SGA were calculated using a logistic regression model, with the 20–24-year age group as reference.

**Results:**

We analyzed 38,412 singleton primiparous pregnancies. The aORs of all outcomes increased in parallel with each maternal age group >30 years. The aORs of PTB before 37 and 34 weeks, LBW <2,500 g, LBW <1,500 g, and SGA in the 30–34-year age group were 1.39 (95% confidence interval [CI], 1.16–1.67), 2.23 (95% CI, 1.45–3.41), 1.34 (95% CI, 1.18–1.53), 2.30 (95% CI, 1.35–3.94), and 1.24 (95% CI, 1.05–1.46), respectively.

**Conclusion:**

The present study showed that higher maternal age (>30 years) at the first childbirth was an independent risk factor for PTB, LBW, and SGA.

## INTRODUCTION

The prevalence of advanced maternal age pregnancy, defined as pregnancy at ≥35 years, is increasing in developed countries. In Japan, from 1990 through 2015, advanced maternal age pregnancies increased from 7.7% to 29.0%, and the mean maternal age at the first childbirth also increased from 27.0 to 30.7 years.^1^ These trends, which have also been observed in other developed countries, may be a reflection of progress in perinatal management, advances in assisted reproductive technology, and an increasing number of women pursuing higher education and careers.^2^ In addition, there has also been an increase in the rate of preterm birth (PTB) before 37 weeks (4.5% to 5.6%) and low birth weight (LBW) neonates <2,500 g (6.5% to 9.5%) and <1,500 g (0.53% to 0.75%) during the same period.

It is widely recognized that advanced maternal age is related to several obstetric complications. PTB, which could cause LBW in a neonate, is one of the major complications leading to significant neonatal morbidity and mortality. However, few studies have examined the effect of delaying first child birth maternal age on PTB, LBW neonates, or small-for-gestational-age (SGA) neonates using nationwide study while simultaneously accounting for confounding factors.^3^^–^^8^

With the increase in first childbirth maternal age, it is essential for obstetric care providers to investigate the precise risk of delayed first childbirth to manage specific risk groups effectively. Thus, we aimed to examine the effect of first childbirth maternal age on PTB, LBW, and SGA, after adjusting for confounding factors using data from a Japanese nationwide cohort study.

## MATERIALS AND METHODS

### Study design

In the present study, we used the data of the Japan Environment and Children’s Study (JECS), which is a nationwide and government-funded birth cohort study^9^ that was started in January 2011 to investigate the effects of environmental factors on children’s health. The eligibility criteria for JECS participants (expecting mothers) were as follows: (1) residing in the study areas at the time of recruitment and expected to reside continually in Japan for the foreseeable future, (2) the expected delivery date should be between August 1, 2011 and mid-2014, and (3) capable of participating in the study without difficulty (ie, they must be able to comprehend the Japanese language and complete the self-administered questionnaire).

The target recruitment rate was more than 50% of all eligible mothers. Either or both of the following two recruitment protocols were applied: (1) recruitment at the time of the first prenatal examination at cooperating obstetric facilities and/or (2) recruitment at local government offices issuing pregnancy journals, namely the *Mother-Child Health Handbook*, which is given to all expecting mothers in Japan before receiving municipal services for pregnancy, delivery, and childcare. Written informed consent was obtained from all participating women.

The JECS protocol was reviewed and approved by the Ministry of the Environment’s Institutional Review Board on Epidemiological Studies and by the Ethics Committees of all participating institutions (The National Center for Child Health and Development, Hokkaido University, Sapporo University, Asahikawa Medical College, Japanese Red Cross Hokkaido College of Nursing, Tohoku University, Fukushima Medical University, Chiba University, Yokohama City University, University of Yamanashi, Shinshu University, University of Toyama, Nagoya City University, Kyoto University, Doshisha University, Osaka University, Osaka Medical Center and Research Institution for Maternal and Child Health, Kyushu University, University of Occupational and Environmental Health, Kumamoto University, University of Miyazaki, and University of Ryukyu). The JECS was conducted in accordance with the Helsinki Declaration and other nationally valid regulations and guidelines.

### Data collection

Data for the current analysis used the data set released in June 2016 (data set: jecs-ag-20160424). In this data set, we used three types of data: (1) T1, obtained from a self-reported questionnaire collected during their first trimester (the first questionnaire) that included questions regarding maternal medical background; (2) T2, obtained from a self-reported questionnaire collected during their second/third trimester (second questionnaire) that included partner lifestyle and socioeconomic status; (3) M0, included obstetrics outcomes, such as gestational age, birth weight, and weight of placenta, collected from medical records provided by each subject’s institution; and (4) maternal blood sample, collected during the first trimester. We excluded cases with inadequate data, multiple pregnancies, teenage mothers, multipara, or delivery before 22 weeks. We excluded subjects <20 years based on the rule of JECS. Similar to advanced maternal age, lower maternal age is thought to be a risk factor for both PTB and LBW.^10^

### Maternal age, obstetrics outcomes, and confounding factors

Maternal age at delivery was obtained from M0 data and then categorized into five age groups: 20–24, 25–29, 30–34, 35–39, and ≥40 years. Obstetric outcome obtained from M0 data included gestational age at birth and birth weight. PTB was classified into two categories: before 37 weeks and before 34 weeks. LBW was classified into two categories: LBW <2,500 g and LBW <1,500 g. SGA was defined as a birth weight below −1.5 standard deviation (SD) corrected for gestational age and sex according to the “New Japanese neonatal anthropometric charts for gestational age at birth”.^11^ The following items were used as confounding factors: maternal smoking status and the Kessler 6-item psychological distress scale (K6) (T1 data), as well as annual household income and education state of mother (T2 data). Maternal participants were requested to provide information about their smoking status: “kept smoking during pregnancy,” “never smoked,” “quit smoking before pregnancy,” and “quit smoking during early pregnancy”. The maternal participants who chose “kept smoking during pregnancy” were classified into the smoking category and the others into non-smoking. We used the Japanese version of the K6 to measure psychological distress in their first trimester. The K6 is a self-administered questionnaire that consists of six questions evaluating depressive state and anxiety on a scale from 0 (little to no depression or anxiety) to 4 (high levels of depression or anxiety). The K6 score is a continuous variable determined by summing six questions, with a possible total score ranging from 0 to 24. A patient with a K6 score ≥13 was defined as having psychological stress.^12^^,^^13^ Annual household income was categorized into four levels (<2,000,000, 2,000,000–5,999,999, 6,000,000–9,999,999, and ≥10,000,000 JPY). The educational state of the mother was categorized into four groups (junior high school: <10, high school: 10–12, professional school or university: 13–16, and graduate school: ≥17 years). The inclusion criteria of confounding factors for this study were determined by clinical importance (ie, those believed to be related to present obstetric complications).^3^^–^^8^

### Statistical analysis

Characteristics were summarized according to the maternal age group. One-way analysis of variance was used to compare the continuous variables between each group and a chi-square test was used to compare the categorical variables. Adjusted odds ratios (aORs) and 95% confidence intervals (CIs) for PTB, LBW, and SGA were calculated using a multiple logistic regression model. The odds ratios were adjusted for maternal education, maternal smoking status, K6 score at the first trimester (continuous), and household income. We accomplished this by using dummy variables for each age group using the 20–24-year age group as the reference.

SPSS version 21 (IBM Corp., Armonk, NY, USA) was used for the statistical analyses. A *P*-value <0.05 indicated statistical significance.

## RESULTS

**Figure 1.**
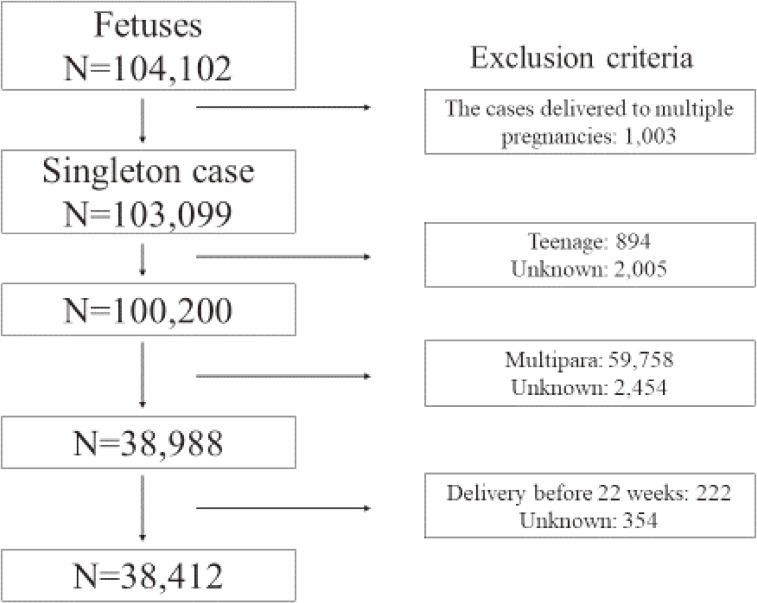
Study enrollment flowchart

The total number of fetal records who delivered from 2011 to 2014 in the JECS was 104,102. After applying our inclusion criteria, 38,412 participants were eligible for the present study (Figure 1). The study consisted of 5,524 births to mothers aged 20–24 years, 13,065 births to mothers aged 25–29 years, 11,992 births to mothers aged 30–34 years, 6,381 births to mothers aged 35–39 years, and 1,450 births to mothers aged ≥40 years.

### Maternal medical and socioeconomic background and obstetric outcomes

Table 1 summarized the maternal medical and socioeconomic background and obstetric outcomes according to maternal age group. Total incidence of PTB before 37 and 34 weeks, LBW <2,500 and <1,500 g, and SGA were 5.3%, 1.4%, 10.2%, 0.9%, and 5.6%, respectively. The prevalence of these outcomes increased along with the maternal age group. The mean weight of the placenta decreased along with the maternal age group.

**Table 1.** Basic information according to maternal age

	Total (*n* = 38,412)	20–24 years (*n* = 5,524)	25–29 years (*n* = 13,065)	30–34 years (*n* = 11,992)	35–39 years (*n* = 6,381)	⩾40 years (*n* = 1,450)	*P*
**Maternal medical background**							
K6 score at the first trimester, mean (SD)	3.9 (4.0)	4.8 (4.5)	4.1 (4.0)	3.7 (3.8)	3.4 (3.6)	3.1 (3.3)	<0.001^a^
K6 score ≥13 at the first trimester, %	3.9	6.9	4.3	3.1	2.7	1.3	<0.001^b^
Smoked during pregnancy, %	3.6	7.2	3.5	2.8	2.9	2.4	<0.001^b^
**Socioeconomic background**							
Annual household income, %							
<2,000,000 JPY	5.2	16.2	4.7	3.0	2.9	2.9	<0.001^b^
2,000,000–5,999,999 JPY	66.0	73.2	71.0	63.1	58.6	54.3	
6,000,000–9,999,999 JPY	24.2	8.4	21.5	28.9	31.1	32.5	
≥10,000,000 JPY	4.5	2.2	2.8	5.1	7.4	10.3	
Maternal education, %							
<10 years	3.1	9.7	3.1	1.4	1.3	1.4	<0.001^b^
10–12 years	29.1	53.9	27.5	22.2	24.3	28.7	
13–16 years	42.9	30.9	44.2	44.2	46.8	47.5	
≥17 years	24.9	5.5	25.2	32.2	27.7	22.4	
**Obstetric outcome**							
Gestational age, weeks, mean (SD)	39.0 (1.8)	39.1 (1.6)	39.1 (1.6)	39.0 (1.8)	38.8 (2.0)	38.7 (2.0)	<0.001^a^
PTB before 37 weeks, %	5.3	4.3	4.5	5.6	6.7	8.3	<0.001^b^
PTB before 34 weeks, %	1.4	0.9	1.0	1.5	1.9	2.6	<0.001^b^
Birth weight, g, mean (SD)	2,980 (439)	3,001 (412)	2,991 (415)	2,979 (447)	2,952 (476)	2,923 (504)	<0.001^a^
LBW <2,500 g, %	10.2	8.4	9.1	10.4	12.8	14.7	<0.001^b^
LBW <1,500 g, %	0.9	0.6	0.7	1.0	1.4	1.8	<0.001^b^
SGA, %	5.6	4.9	5.1	5.7	6.7	7.6	<0.001^b^
Weight of placenta, g, mean (SD)	556 (121)	561 (138)	557 (111)	554 (122)	554 (122)	547 (124)	<0.001^a^

JPY, Japanese yen; LBW, low birth weight; PTB, preterm birth; SD, standard deviation.

^a^*P*-value, one-way analysis of variance.

^b^*P*-value, chi-square test.

### Risk of each obstetric complication in the first live born

Table 2 summarizes the aORs of obstetric complication according to maternal age group. The aORs of all outcomes increased in parallel with each maternal age group >30 years. The risk of LBW <2,500 g was also increased in the 25–29-year age group compared with the 20–24-year age group. When we compared the aORs between PTB before 37 and 34 weeks and between LBW <2,500 and <1,500 g among the same age group, higher aORs were observed in PTB before 34 weeks and LBW <1,500 g in all age groups.

**Table 2.** Relation between maternal age and preterm birth, low birth weight, and small for gestational age

Maternal age, years	PTB				LBW				SGA	
	
	<37 weeks		<34 weeks		<2,500 g		<1,500 g			
				
	aOR	95% CI	aOR	95% CI	aOR	95% CI	aOR	95% CI	aOR	95% CI
20–24	Ref	Ref	Ref	Ref	Ref	Ref	Ref	Ref	Ref	Ref
25–29	1.08	0.91–1.30	1.46	0.94–2.25	1.17	1.03–1.33	1.40	0.81–2.43	1.12	0.95–1.31
30–34	1.39	1.16–1.67	2.23	1.45–3.41	1.34	1.18–1.53	2.30	1.35–3.94	1.24	1.05–1.46
35–39	1.63	1.35–1.98	2.93	1.88–4.56	1.68	1.46–1.93	3.09	1.78–5.38	1.46	1.22–1.74
≥40	2.14	1.66–2.76	4.39	2.59–7.43	2.07	1.71–2.51	4.34	2.23–8.41	1.69	1.32–2.18

aOR, adjusted odds ratio; CI, confidence interval; LBW, low birth weight; PTB, preterm birth; Ref, reference.

aOR was calculated by logistic regression analysis, using maternal education, maternal smoking status, K6 score at the first trimester, and household income.

## DISCUSSION

Although maternal age >35 years is widely accepted as advanced, the present study found that the risk of PTB, LBW, and SGA in the first pregnancy increased stepwise in subjects >30 years after adjusting for several confounders.

Our results indicate that the effect of advanced maternal age on pregnancy outcome is consistent with recent studies.^14^^–^^16^ This finding is also similar to that of a previous study using the same primiparous model,^4^ which reported that the aORs of LBW <2,500 g and PTB before 37 weeks were significantly increased in the 30–34-, 35–39-, and ≥40-year age groups. However, since we analyzed a larger number of participants and included psychological distress, measured using the K6 score, in logistic model than in that study, the aOR of PTB before 37 weeks and LBW <2,500 g in each age group was similar but the aOR of LBW <1,500 g became higher in our study. Furthermore, compared to previous primiparous model study, we also examined the risk of more severe PTB and LBW (ie, PTB before 34 weeks and LBW <1,500 g). As a result, we found that the risk of PTB before 34 weeks and LBW <1,500 g was more relevant among each age category. With regard to maternal age, we used maternal age as categorical variable rather than continuous one to estimate the risk of each age category, with the 20–24-year age group as reference. We believe that using maternal age as a categorical variable is more practical because it would be able to provide more appropriated support to expectant mother for each age category group with consideration of each age group’s risk factor.

The reasons why aging itself causes obstetric complications, such as SGA, are not fully understood. SGA caused by aging may be attributable to changes in the vasculature, which could lead to sclerotic lesions in the myometrial arteries.^17^ Some authors have reported that poor oxygen exchange is associated with advanced maternal age, which might lead to an increased risk of SGA.^18^ Placental dysfunction related with obstetric complications, including preeclampsia, SGA, and placental abruption, are associated with advanced maternal age.^19^ Significant differences in the weight of the placenta between the maternal age groups in the present study may support these hypotheses. PTB has the same endpoint, consisting of two clinical subtypes—namely spontaneous PTB and medically indicated PTB—which is conducted for cases of SGA or preeclampsia.^8^ The increase in PTB due to maternal aging may be a result of an increase in medically indicated PTB, such as SGA, as we have shown in this study. The present study did not categorize PTB into spontaneous or medical indication; therefore, further study is required to examine whether aging itself is related to another obstetric complication that leads to medically indicated PTB.

Our study has limitations that need to be considered. First, maternal smoking status relied on a self-reported questionnaire instead of objective measurement and was assessed only during their first trimester. A questionnaire-based study design can sometimes lead to an underestimation of the maternal smoking rate.^20^ However, other data on the maternal background and obstetric outcome from the M0 data, which were based on medical records collected prospectively by physician, midwives, nurses, and trained research coordinators, are likely to be relatively accurate. Second, Khalil et al^21^ reported that maternal ethnicity was related to adverse pregnancy outcomes; however, according to the JECS recruitment protocols, almost all participants in our study were Japanese women. Therefore, our results did not consider the maternal ethnicity, and the findings may not be applicable to other ethnicities.

Despite these limitations, there are several strengths to the present study. JECS is the first large nationwide population-based study in Japan combining medical records and biological samples managed by the Japanese government with meticulous attention to data precision. JECS also included various types of evaluation items for each pregnant woman. A preliminary report of approximately 10,000 pregnant women recruited during the first year of the JECS study and the baseline profile of JECS showed no significant differences in the distribution of maternal age between the JECS participants and the general population.^22^^,^^23^

In a clinical setting, one of the most reliable screening methods that can be easily used to predict PTB is a history of preterm birth.^24^ However, this cannot be applied to primipara cases. Therefore, our study, which assessed the risk of PTB from primiparous data, can contribute toward enhancing preconceptual counseling for delayed-age child-bearing mothers.

In conclusion, our study found that maternal age >30 years is an independent risk factor for PTB, LBW, and SGA after adjusting for several confounding factors. With increasing mean maternal age at first childbirth in Japan, it is very important for obstetric care providers to provide the latest data and proper counseling to women of advanced age expecting to be pregnant for the first time.
